# Christopher Pegge

**Published:** 1822-09

**Authors:** 


On Saturday, the 3d of August, at Oxford, in the 58th year of his age, Sir CHRISTOPHER PEGGE, knight, Regius Professor of Physic at that university.

---

He has been succeeded in his professorship by JOHN KIDD, M.D. whose appointment took place on the 9th of August.

### METEOROLOGICAL JOURNAL.

